# Time-varying causality relationships between trade openness, technological innovation, industrialization, financial development, and carbon emissions in Thailand

**DOI:** 10.1371/journal.pone.0304830

**Published:** 2024-05-31

**Authors:** Nguyen Thi Quy, Nguyen Chi Hai, Ha Thi Thieu Dao

**Affiliations:** 1 University of Economics and Law, Ho Chi Minh City, Vietnam; 2 Vietnam National University, Ho Chi Minh City, Vietnam; 3 Ho Chi Minh Banking University, Ho Chi Minh City, Vietnam; Lahore School of Economics, PAKISTAN

## Abstract

Over the last twenty years, there has been swift growth in industrialization and technological advancements, driving economic progress. Nevertheless, it is inevitable that these sectors will bring about environmental shifts. Thus far, endeavors have been undertaken to assess the influence of industrialization and technological advancements on environmental deterioration. Additionally, the extensive discussion surrounding the impact of financial development, trade openness, and technological innovation on the environment has not yielded conclusive empirical findings. Studies often operate under the assumption of symmetric relationships, potentially leading to biased results. Adding to the discussion on the drivers of carbon neutrality, the time-dependent effects of critical aspects such as financial development and technological innovation should inform meaningful policies for environmental management. This article explores the time-varying causal association between trade openness, industrialization, financial development, technological innovation, and CO2 emissions in Thailand using novel time-varying Granger causality tests. The time-varying causality outcomes demonstrate that the associations change significantly over time, in contrast to the results of Toda-Yamamoto causality. Overall, there exists a bidirectional relationship between industrialization, financial development, trade openness, technological innovation, and CO2 emissions over different time sequences. These outcomes have implications for both policy and research.

## 1. Introduction

Over the past few decades, advancements in human well-being and global economic growth have been accompanied by rapid resource depletion and an increase in CO2 emissions, raising awareness about environmental issues. The Environmental Kuznets Curve (EKC) concept illustrates the trade-off between environmental conservation and economic development. It suggests that as economic growth accelerates, environmental conditions initially deteriorate before improving [1]. The endogenous economic growth theory posits that while increased spending on Research and Development (R&D) can lead to more efficient resource utilization, the impact of technological advancement and financial development on environmental quality, particularly CO2 emissions, remains uncertain [2,3].

Understanding our surroundings and the environment would enable us to embrace the concepts of economic growth and general development without endangering the ecosystem. It also enables us to understand the many sorts of habitats and their consequences for their residentials, which are able to assist them in improving their quality of life through environmental preservation [4–7]. The research will look into the direct and indirect consequences of financial development, technical innovation, trade openness, and industrialization on pollution emissions in Thailand.

The reasons behind considering this country appropriate for our study are as follows: First, recent years have seen rapid economic expansion in Thailand, a developing ASEAN economy rich in natural resources [8]. Thailand’s economy has expanded significantly, and its overall economic growth increased from US$ 401.3 billion to US$536.16 billion between 2015 and 2022 [9]. The rapid development encourages technological innovations, financial development, industrialization, and the usage of natural resources [10]. Second, Thailand has significantly contributed to CO2 emissions because of the combustion of fossil fuels over the last few decades. In addition, Thailand was particularly heavily impacted by the 1997 East Asian recession, which created a significant economic slump and political instability. As a result, it makes sense to look into the dynamic association between financial development, trade openness, technological innovations, industrialization, and environmental quality in Thailand. According to Salman et al.[11], spatial institutional spillover is a mechanism via which a country’s environmental quality can simultaneously extend to its surrounding countries, in addition to having an influence on the nation’s own environmental quality.

There is a lack of research on the analysis of these competing paradigms through the lenses of industrialization, trade openness, financial development, and technological innovations in order to understand a feasible policy framework to harness the positive contributions of these indicators without incurring their accompanying negative impacts on the environment [12–15]. This study aims to address the observed gap by emphasizing policy design that promotes economic growth while acknowledging environmental sustainability without neglecting its challenges, based on the causal association between the variables of interest.

This analysis centers on Thailand and is motivated by the work of Yue et al. [10] on the green innovation and emissions relationship. We employ longer period data, a well-defined empirical framework, and a superior recursive evolving window procedure based on the series integration order. Further, several research have looked at two or more of the variables in this study, but none have looked at all five (financial development, industrialization, technical innovation, trade openness, and CO2) in a single regression. Hence, the inference of the chosen indicators on CO2 emission using time-varying evidence from Thailand, in which they are constructed into dynamic Granger causality tests, has never been considered in any study and so greatly adds to the literature, increasing the novelty of the current study.

Furthermore, it is worth highlighting that the nonlinear relationships between financial development, TEC, industrialization, trade openness, and CO2 emissions in Thailand are inspiring. Causality is time sensitive [16]. To that purpose, throughout the investigation, we utilize both conventional and time-varying Granger approaches to detect the causal effects between the examined variables. The time-varying Granger causality uses a strong econometric approach, skips the pre-processing processes of detrending or differencing, and can pinpoint the beginning and end dates of causal events [17]. Understanding the bidirectional causation might aid in determining the timeline of the relationship. Specifically, our findings illustrate considerable Granger causality between regressors and CO2 emissions, emphasizing the importance of governments and individuals addressing sustainable development challenges.

Put differently, the purpose of the current article is to analyze the time-varying dynamics between financial development, industrialization, technical innovation, trade openness, and environmental quality in Thailand during the sample period from 1991 to 2022. Our work makes three contributions to related literature. First, the majority of earlier investigations relied on traditional causality tests, which assumed that the causal association remained constant across time. Nevertheless, this assumption needs to be modified in light of the existence of fluctuations, structural alterations, and shocks that could have changed how the two-time series interacted with one another. We discover bidirectional Granger causality between the selected variables by employing novel dynamic techniques based on VAR frameworks. By illustrating the temporal variety in the time-varying causality between the investigated variables and emissions in Thailand, we add to the body of current knowledge. Second, financial development and technological innovation have a remarkable influence on reducing emissions. Existing studies [6,14,18–21]. report the significant interplay between technological innovation and CO2 emissions. Our findings add to the body of literature by further examining the influence of financial development and technological innovations on emissions. Third, our results reveal that trade openness, financial development, industrialization, and technological innovations have a more sustained effect on CO2 emissions in Thailand, which provides policy implications for effective environmental management.

The next section highlights the existing literature on the topic. The data and methodology are documented in Section 3. Section 4 represents the empirical results. Finally, the conclusion and policy implications are reported in Section 5.

## 2. Theoretical background and literature review

### 2.1 Carbon emissions and trade openness

Grossman and Krueger [1] introduced the EKC hypothesis based on Kuznets [22], and this was the pioneer study that explored the nexus between trade openness and CO2. Since then, the EKC theory and the interplay between emissions and global trade have been the subject of numerous studies. Copeland and Taylor [23] indicate that trade openness significantly influences emissions through two main channels: the scale effect and the composition effect. The composition effect describes how trade affects how output is distributed among nations, while the impact of commerce on the degree of economic activity is known as the scale effect.

The existing environmental literature also provides studies looking into the associations between trade openness and CO2 with ambiguous results. For example, Hossain [24] reveals that there is a unidirectional short-term nexus from trade openness and GDP to CO2 but no evidence of a long-term causative interplay in NIC countries. Ertugrul et al. [25] suggest that trade openness is an important determinant in CO2 in the long run among developing countries. Nevertheless, Zhang et al. [26] promote the existence of EKC theory and uncover that there is a negative impact of trade openness on environmental degradation in ten industrialized economies.

### 2.2 Carbon emissions and industrialization

Theoretical justifications for why industrialization might have a negative impact on the environment have been studied in the literature [27]. The idea is frequently linked to nations moving to greater use of natural resources and higher energy consumption in an effort to raise their industrial profile. Recently, there has been a rise in national and regional research on the impact of industrialization on CO2. There is a positive correlation between industrialization and carbon emissions when the whole industrial production is taken into account [28–30]. Other research has looked into whether the effects of certain industrialization categories on the environment differ between countries [31–33]. For example, Liu and Bae [34] show that industrialization increases CO2 in China. In Bangladesh, Shahbaz et al [3] uncover that EKC exists between industrialization and CO2, while financial development and trade openness have a positive impact on emissions. In a sample of 44 Sub-Saharan African nations, Mentel et al. [12] discover that the fraction of GDP devoted to industry has a remarkable beneficial influence on CO2 emissions. As per Mahmood et al. [35], both urbanization and industrialization impair the emissions, with industrialization having an inelastic effect and urbanization having an elastic effect on environment in Saudi Arabia. Xu and Lin [4] corroborate the inverted U-shaped nonlinear link between CO2 emissions and industrialization in China’s three regions.

### 2.3 Carbon emissions and financial development

Environmental quality is greatly influenced by financial development. The research points to both beneficial and detrimental environmental implications of financial development. Financial development lowers emissions by giving domestic businesses financial support for eco-friendly technology. By contrast, there are a number of ways that financial development can degrade environmental quality. Supporting local businesses financially can increase their manufacturing, which increases CO2 emissions and degrades the land.

Several articles have examined the practical interactions between financial development and CO2, instead than exclusively focusing on theoretical aspects. As per Lv and Li [5], there may have been a considerable negative overall effect because the significant beneficial direct impact of financial development on CO2 was overshadowed by the considerable negative spillover effect. Khan and Ozturk [18] support the EKC hypothesis for the panel of economies and show that DF mitigates the negative impacts of income, trade openness, and FDI on pollution emissions. According to Khezri et al. [36], financial growth indicators are becoming more relevant and are stimulating a rise in CO2 in 31 Asia-Pacific economies. Using a global sample of 100 nations, Bui [19] confirms the beneficial direct influence of financial development on environmental degradation. Similarly, Kihombo et al. [37] document that for the WAME economies, financial development confirms the environmental Kuznets curve phenomena and adds to environmental degradation.

Furthermore, Xu et al. [38] focus on Saudi Arabia and suggest that environmental quality is deteriorated, and CO2 emissions are increased by financial development. The asymmetric relationship between financial development and the environment is supported by Majeed et al. [13], since negative shocks to financial growth have a dramatic short- and long-term influence on CO2 emissions in Malaysia.

### 2.4 Carbon emissions and technological innovations

Manufacturing or production technology innovation is referred to as technological innovation. It typically takes two forms: the inventive use of pre-existing technology and the creation of new technology [6,39]. One of the main approaches to solving environmental issues and promoting sustainable development is technological innovation [40]. Systematic analysis of the influencing mechanisms is required since technological innovation has both direct and moderating effects on the decrease of CO2 emissions.

According to Ma et al. [41], China’s carbon-abatement strategy is made easier by increased energy investments, technical innovation, renewable energy consumption, spending on carbon emission taxes and research and development. These factors all work together to reduce CO2. Similarly, Wang and Zhu [42] reveal that innovation in renewable energy technology helps this nation reduce its CO2 emissions. As per Adebayo et al. [43], renewable energy utilization contributes to reduce CO2, whereas trade openness, technical innovation, and GDP all contribute to CO2 emissions in Portugal. Jian and Afshan [14] document that green finance and technologies enhance environmental quality in G10 economies. Bilal et et al. [15] indicate that across all regions (Central Asia, South Asia, OBOR, Europe, MENA, East and Southeast Asia), the relationship between technological innovation and CO2 emissions is statistically significant and negative. However, Chen and Lee [44] suggest that advanced technology has no significant impact on CO2 globally.

In the context of Thailand, Kongbuamai et al. [8] confirm that trade openness and economic growth positively impact the ecological footprint in the long term. In the same vein, Salman et al. [45] disclose a positive interaction between trade openness and CO2 during the periods of 1990–2016. Le et al. [46] unveil that increased trade openness results in improved CO2 emissions for global economies. Wenlong et al. [47] reveal that technological innovations and trade openness have a detrimental influence on CO2 in Asian economies.

In order to fully realize the potential of renewable energy sources, a significant amount of electricity must be generated by them by 2050, according to research done by Kumar [48] on the energy mix potential of two Southeast Asian nations, Indonesia and Thailand. In this sense, carbon dioxide emissions in these nations have declined by 81% and 88%, respectively. Adebayo et al. [49] find that a positive intercorrelation exists between CO2 and financial development in Thailand. Saidi and Mbarek [50] point out that financial development lowers environmental damage and has a long-term negative influence on CO2. Similarly, Yue et al. [10] discover that green innovation and tourism, like foreign investments, reduce environmental harm by lowering CO2 emissions and that green tech innovation enhances environmental quality by lowering CO2.

Numerous scholars have investigated the relationship between the selected enviro-economic variables over the years; nevertheless, because they have used different methodologies, data (time series and panel), study lengths, and nations examined, their conclusions are not entirely consistent. On the other hand, by using time-varying causation, our analysis initiates a new discussion regarding the relationship between various economic indices. Consequently, by applying Granger causality based on time-series features, the research closes the gap in the literature. Moreover, no study in the reviewed literature looked at this relationship for Thailand’s situation using these combined economic indicators.

## 3. Methodology

To present the time-varying Granger causality, we start with setting up the following unrestricted VAR (k) model:

$$z_{t}=\varphi_{0}+\varphi_{1}z_{t-1}+\cdots +\varphi_{k}z_{t-k}+\varepsilon_{t}$$ (1)

or equivalently presented in a multivariate regression form:

$$z_{t}=\psi_{x_{t}},t=\bar{1,T}$$ (2)

where $z_{t}=(z_{1t},z_{2t}),x_{t}={\left(1,z_{t-1}^{,},z_{t-2}^{,},\cdots ,z_{t-k}^{,}\right)}^{,}$ and $\psi_{2\times (2p+1)}=[\varphi_{0},\varphi_{1},\cdots ,\varphi_{k}]$

Let $\hat{\psi }$ denote the OLS estimator of *ψ*, $\hat{\Omega }=\frac{1}{T}\sum_{t=1}^{T}{\hat{\varepsilon }}_{t}{\hat{\varepsilon }}_{t}^{,}$ with ${\hat{\varepsilon }}_{t}=z_{t}-\hat{\psi }x_{t}$ and $X_{t}^{'}=[x_{1},\ldots ,x_{T}]$ is the matrix of the observations on the independent variables in (1).

In this case, we are able to test the null hypothesis that *y*_2*t*_ does not Granger cause to *y*_1*t*_. The Wald test for such constraints can be formulated as follows:

$$W={\left[Rvec(\hat{\Pi })\right]}^{'}[R(\hat{\Omega }⊗{\left(X^{'}X\right)}^{-1})R^{'}][Rvec(\hat{\Pi })]$$ (3)

where $vec(\hat{\Pi })$ denotes the 2(2*k*+1)×1 coefficients of $\hat{\Pi }$ and R is the *k*×2(2*k*+1) selection matrix. There exist *p* coefficients on the lagged values of *z*_2*t*_ given in (1).

The Wald test provides the foundation for identifying causal relationships. There is a significant shift in the causal relationship shown if the Wald statistical sequence exceeds the relevant critical value. The first observation whose test statistic value exceeds its corresponding critical values is identified as the start date of a change in the causal relationship.

The real-time-varying causality test, presented by Shi et al. [16,51], is based on a recursive evolving algorithm and employs supremum (sup) Wald statistic sequences. Let *f*_*e*_ and *f*_*f*_ represent the starting and ending points of the causal relationship. These are the first observations where the test statistic exceeds or falls below the crucial value. The Wald statistic over *f*_*w*_ = *f*_2_−*f*_1_≥*f*_0_ is given by $W_{f_{2}}(f_{1})$ and the sup Wald statistic is denoted by:

$$SW_{f}(f_{0})=\frac{sup}{(f_{1},f_{2})\in \wedge_{0,}f_{2}=f}\{W_{f_{2}}(f_{1})\}$$ (4)

where $\wedge_{0}=\{(f_{1},f_{2}):0<f_{0}+f_{1}\leq f_{2}\leq 1,and\;0\leq f_{1}\leq 1-f_{0}\}$ for some minimal sample size *f*_0_ ∈ (0, 1) in the regressions

For a basic switch example, the dating rules are defined by based on the recursive developing algorithm as follows:

$$\hat{f_{e}}=\frac{inf}{f\in [f_{0},1]}\{f:SW_{f}(f_{0})>scv\}\;and\;\hat{f_{f}}=\frac{inf}{f\in [\hat{f_{e},1}]}\{f:SW_{f}(f_{0})<scv\}$$ (5)

where SCV is the subsequent critical values of the *SW*_*f*_ statistics.

**Fig 1:**
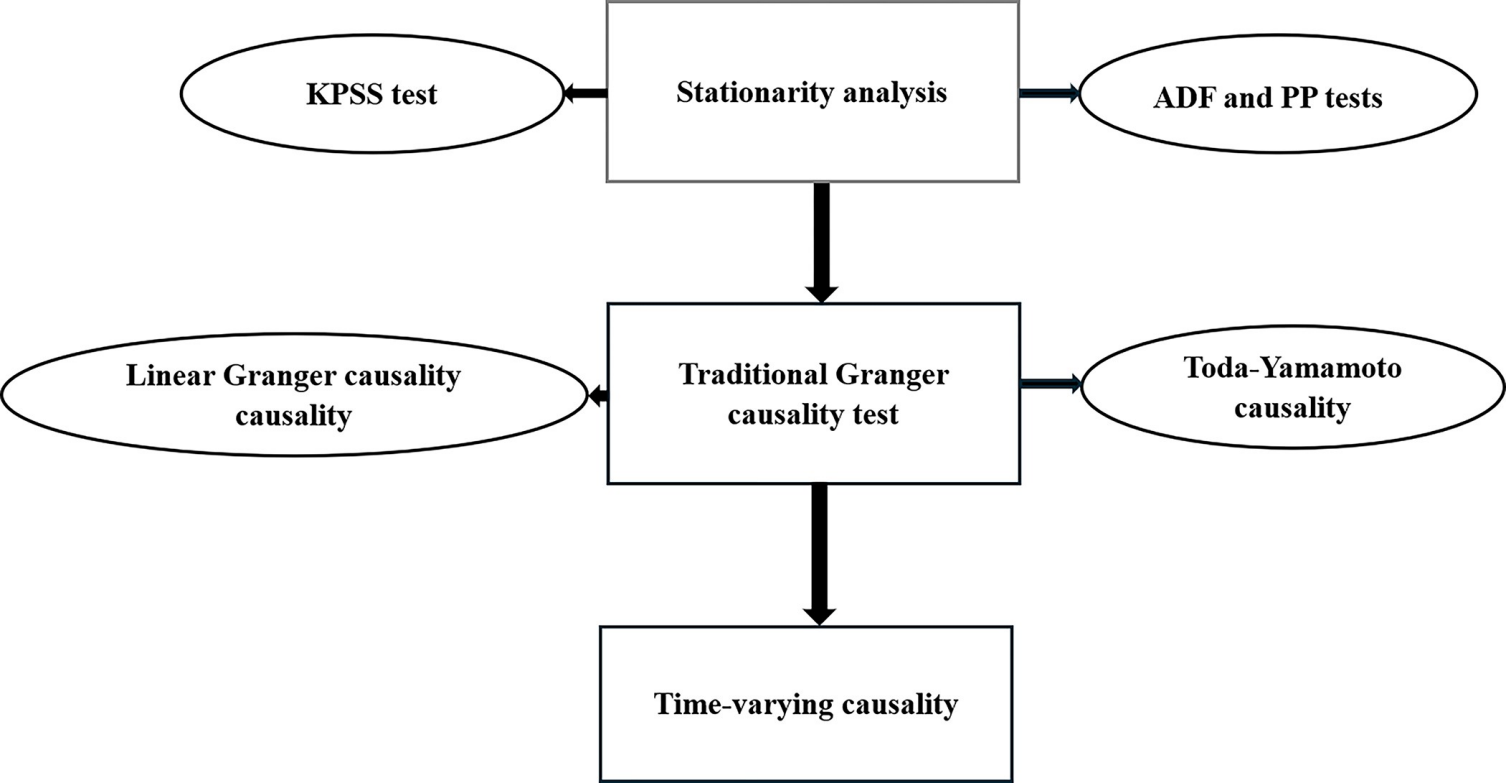
Stepwise econometric methodology.

Fig 1 summarises the stepwise econometric methodology.

## 4. Data

The present paper gives an empirical investigation of the time-varying causality between financial development (DF), technological innovations (TEC), industrialization (IND), trade openness (TO), and CO2 emissions (CO2) in Thailand. Based on the previous studies, it appears that DF and TEC significantly decrease the level of CO2, while TO and IND are the primary sources of CO2 emissions. Thailand is an emerging nation that is rapidly industrializing and developing economically, which is improving the quality of the environment. As a result, the present paper chooses these indicators to analyze their effects on carbon emissions. The annual data are converted into monthly time series by using the quadratic match sum approach and a widely recognized interpopulation methodology to expand the number of observations in the data set [52,53]. As a result, 384 observations in total, a suitable duration for this study, have been made. To solve the variable variance issue, all series are logarithmically converted. Time series data from 1991 to 2022 for Thailand are gained from the World Development Indicator [9] except TEC, which is obtained from the OECD website [54]. For further detail, Table 1 offers the details of the indicators following their description, measuring units, and sources.

**Table 1:** Indicators with description, measuring units, and sources.

Abbreviation of variables	Description	Measuring units	Data sources
CO2	CO2 emissions	Metric tons per capita,	WB (2023)
DF	Financial development	Domestic credit to private sector by banks (% of GDP)	WB (2023)
TEC	Technological Innovation	Patents on environment technologies	OECD (2023)
TO	Trade openness	The ratio of total exports and imports to GDP (% GDP)	
IND	Industrialization	Industry (including construction), value added (% of GDP)	WB (2023)

**Fig 2:**
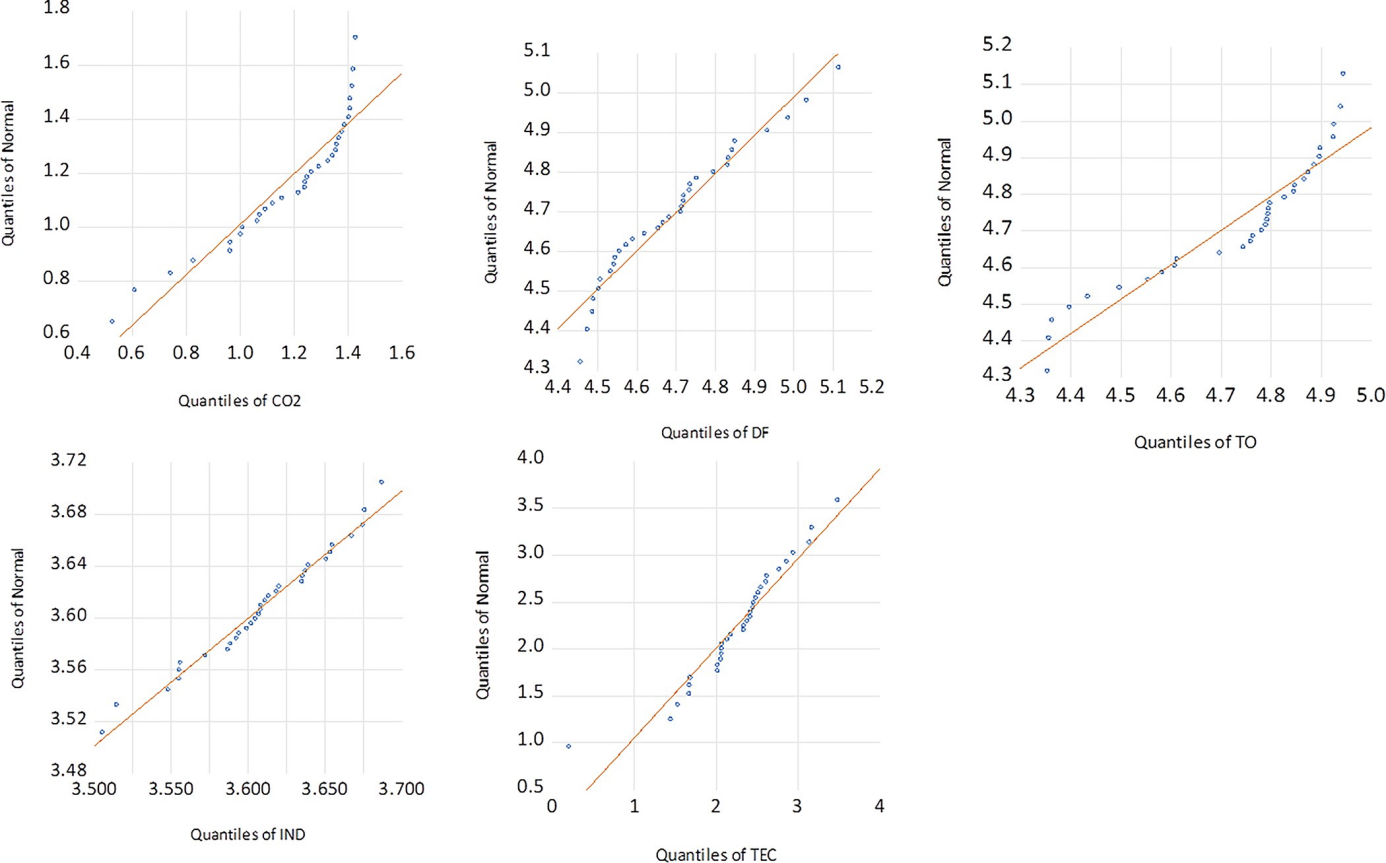
Q-Q plots.

Table 2 displays the descriptive statistical analysis for the study elements. As can be seen that trade openness is the highest in terms of average values, whilst CO2 has the least mean value. Moreover, IND, TO, and DF have low volatility as their data values are clustered around the mean, while CO2 and TEC have a high standard deviation. Additionally, all variables are negatively skewed except for DF since they have a large number of data values on the left side of their distributions, and their right tails are long. The distributions of these indicators are peaking as their kurtosis is less than 1. These outcomes support the Jarque-Bera test that they are normal except for CO2 and TEC. More importantly, an illustrated depiction of the variables is also preferred to convey vital information about their normality (see **Fig 2**). The diagram indicates that there is an unusual dispersion of the parameter data, as the distributions diverge from a straight line.

**Table 2:** Descriptive statistics.

Variables	CO2	DF	IND	TO	TEC
Mean	1.177005	4.693443	3.608389	4.723564	2.273359
Median	1.245404	4.697169	3.608117	4.790998	2.359212
Max	1.428680	5.115017	3.686921	4.944759	3.484926
Min	0.527607	4.456975	3.505216	4.353449	0.198851
Std. Dev	0.243771	0.172523	0.044864	0.188255	0.610385
Skewness	-1.096460	0.611412	-0.372604	-0.800144	-0.967111
Kurtosis	3.433968	2.681180	2.756566	2.347166	5.633073
Jarque-Bera	6.662968	2.129263	0.819461	3.982815	14.23239
Probability	0.035740	0.344855	0.663829	0.136503	0.000812
Observations	384	384	384	384	384

**Fig 3:**
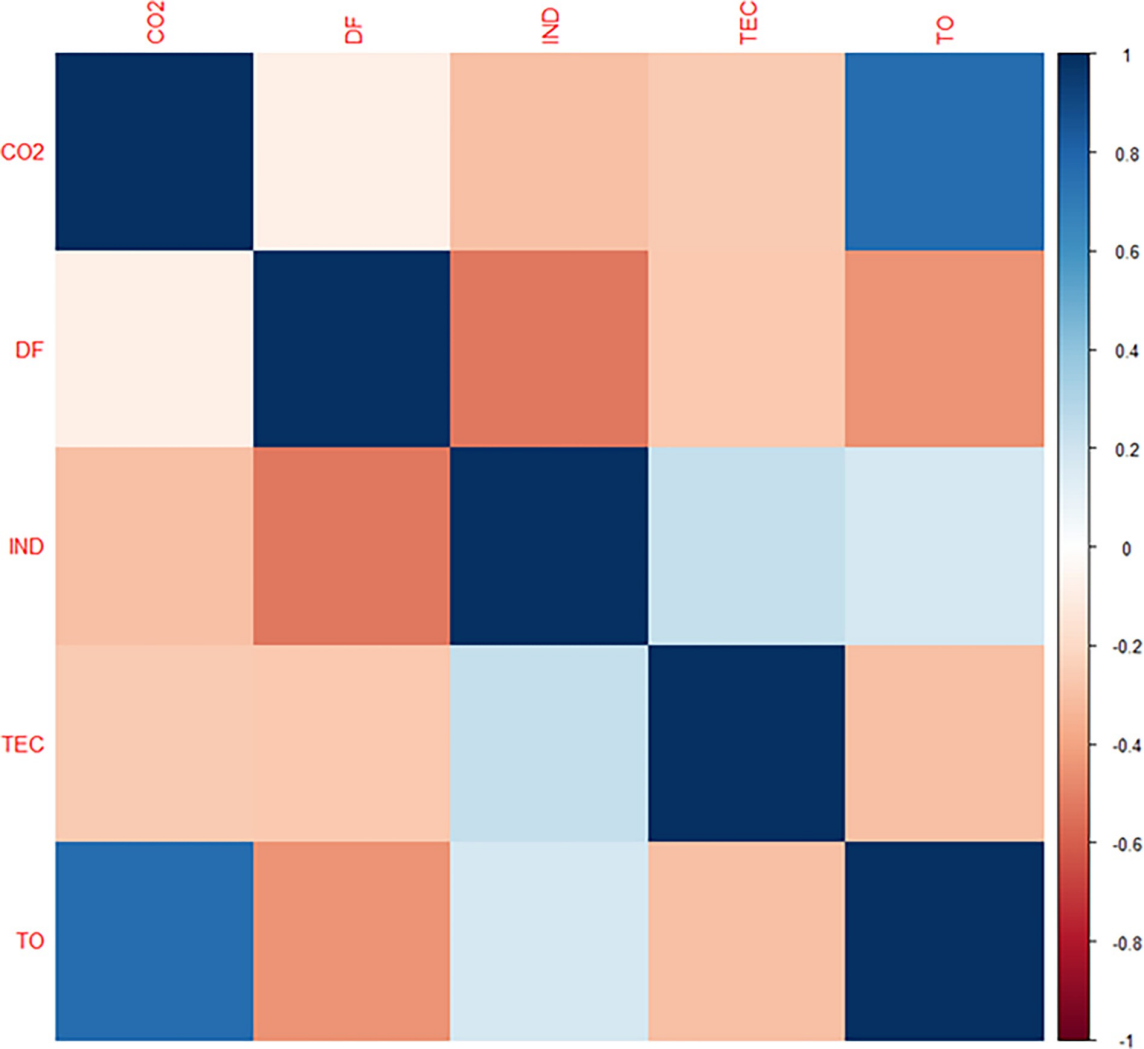
Heatmap correlation matrix.

**Fig 3** illustrates the pairwise correlations between the examined variables. CO2 and TO have the strongest pairwise Pearson correlation, while CO2 and DF have the lowest. In addition, it is worth noting that most of the pairwise coefficients are statistically significant, suggesting that there exists a remarkable interaction between the indicators under investigation. As a result, there is clear evidence of potential associations that would be looking into using more robust econometric models.

## 5. Empirical results and discussion

### 5.1 Unit root analysis

To examine the time-varying causality between TEC, TO, DF, IND, and CO2 emissions, we first test for a unit root in all variables in Thailand. By doing this, we use ADF, PP, and KPSS traditional unit root tests to take into account the stationarity characteristics of the data series. Table 3 summarizes the results of the ADF, PP, and KPSS tests and show that at their level, all indicators are non-stationary, and the null hypothesis of stationary cannot be rejected. All variables are found to be stationary at their first differences, which means that the maximum order of integration for traditional Granger and time-varying causality test is *d* = 1.

**Table 3:** Unit root test results.

	PP		ADF		KPSS		Order of integration
	Intercept and trend		Intercept and trend		Intercept and trend		
Variables	Level I(0)	First Diff I(1)	Level I(0)	First Diff I(1)	Level I(0)	First Diff I(1)	
CO2	-2.348	-3.825***	-2.187	-3.892***	0.694***	0.495**	I(1)
DF	-2.069	-2.786**	-1.774	-2.762**	0.117	0.146**	I(1)
IND	-1.362	-5.158***	-1.180	-5.162***	0.280	0.500***	I(1)
TO	-1.829	-5.332***	-1.872	-5.332***	0.154	0.467**	I(1)
TEC	-3.197*	-8.110***	-3.264	-7.888***	0.097	1.731*	I(1)

Notes: The levels of statistical significance are represented by

***, **, and *, which correspond to 1%, 5%, and 10% significance levels, respectively.

Before proceeding with conventional Granger causality tests and time-varying Granger causality tests, the lag lengths of the variables under consideration must be determined. The VAR lag selection criteria was used to determine the best lag duration. The appropriate lag length based on Akaike information criteria is shown in **Table 4**.

**Table 4:** Optimal lag length for pairs of DF, IND, TO, TEC and CO2.

Pairs of variables	Lag length	
	AIC	BIC
CO2-DF	4	12
CO2-IND	4	12
CO2-TO	5	12
CO2-TEC	4	12

### 5.2 Toda-Yamamoto causality analysis

After identifying the stationarity of the indicators, we now carry out and represent the outcomes of the linear Granger and Toda-Yamamoto causality tests between DF, IND, TO, TEC, and carbon emissions in Thailand. **Table 5** shows the outcomes of the Granger causality test [55]. We find evidence of a unidirectional causality running from CO2 to DF, which suggests that DF would be an effective driver of CO2 emissions in Thailand. However, the null hypothesis has been accepted because the p-values of the F-test are greater than the 10% significance level for the rest of the pairs.

**Table 5:** Granger causality test results.

Null hypothesis	F-test	p value
*CO*2→*DF*	3.693	0.025
*DF*→*CO*2	2.255	0.106
*CO*2→*TO*	1.225	0.294
*TO*→*CO*2	0.284	0.752
*CO*2→*IND*	1.014	0.363
*IND*→*CO*2	0.185	0.830
*CO*2→*TEC*	0.110	0.895
*TEC*→*CO*2	0.675	0.509

We apply the *Akaike information criterion* (AIC) in choosing the optimal lag length for the regressions of VAR models. It is clear from Table 6 that there is a bidirectional causality between financial development and CO2 emissions at the 5% significance level in this country. Furthermore, we find a unidirectional relationship running from industrialization to CO2 at the 10% significance level. On the other hand, there is no causal association between TO, TEC, and CO2, which implies the validity of the neutrality hypothesis in Thailand, which supports the Granger results.

**Table 6:** Results of Toda-Yamamoto test for the entire sample period.

Null hypothesis	MWALD	p value
*CO*2→*DF*	**4.510**	0.004
*DF*→*CO*2	**7.387**	0.024
*CO*2→*TO*	0.569	0.752
*TO*→*CO*2	2.450	0.293
*CO*2→*IND*	0.370	0.830
*IND*→*CO*2	**5.029**	0.062
*CO*2→*TEC*	0.221	0.895
*TEC*→*CO*2	1.350	0.509

However, we believe the traditional Granger and Toda-Yamamoto causality results are erroneous and inconsistent in this research since all of our indicators are sensitive to a variety of endogenous and exogenous political, financial, and economic dynamics and innovations. More so, it is commonly known that Thailand has undergone fundamental changes during the previous decades [47,50]. More specifically, the recent dynamisms, shocks, and structural changes may have had a favorable or unfavorable impact on the behavior of the time series variables. Put differently, unknown change points in the relationship are not taken into consideration by traditional Granger causality testing, while the causal associations between the selected variables might co-move through time, which is able to lead to unreliable test outcomes from Toda-Yamamoto causality tests [56]. As a result, the assumption of time-invariant causality between these variables during the sample period would offer inaccurate outcomes and result in the wrong policy prescription.

We therefore execute the time-varying Granger causality test to avoid spurious results. This method allows for time heterogeneity in the causal relationships and is critical for detecting any potential change points in the causal relationship between TEC, TO, IND, DF, and CO2 emissions.

### 5.3 Time-varying Granger causality analysis

**Fig 4:**
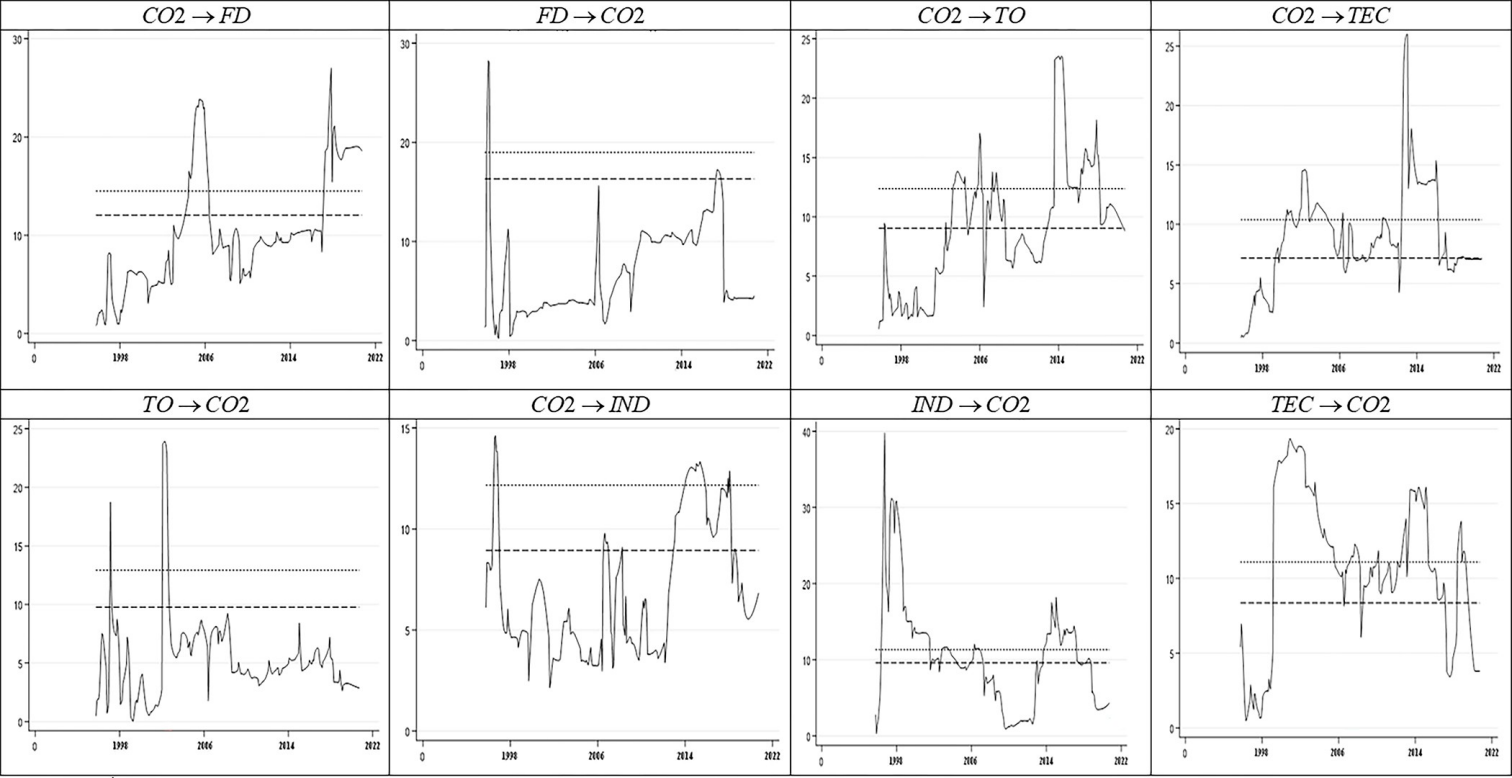
Time-varying Granger causality between IND, TO, DF, TEC, and CO2 emissions for Thailand. Notes: (..) denotes the 10% critical value and (—) denotes the 5% critical value sequences.

In this section, we address the empirical findings that demonstrate the time-varying causality between the selected macroeconomic variables and CO2 emissions. We utilized the TGVC Stata module to estimate the bivariate VAR and run the time-varying Granger causality tests [57]. We went through several procedures, including determining the order of integration, selecting the VAR model, conducting full-sample causality tests, and conducting time-varying causality tests. The findings of the dynamic Granger causality tests are documented in Fig 4. A considerable causality can be determined in the LA-VAR framework for all subsample regressions if the Wald statistic exceeds the related critical value. Because the VAR model under discussion contains variables I(1), the time-varying causation is obtained from a LA-VAR model with d = 1. Lag orders, like Shi et al. [51], are chosen using BIC with a maximum length of 12 (Table 4).

The graphs of the recursive evolving window test statistics and the 5% and 10% critical value sequences for Thailand, respectively. These plots illustrate the test statistics testing the null hypothesis that the selected macroeconomic indicators do not cause CO2 emissions, and the opposite case. Immediately, it is clear that the direction and degree of causation alter over time in all interactions. The null hypothesis of no causality is rejected when the test static sequence exceeds both 5% and 10% critical values.

Fig 4 reports a graphical representation of the findings of dynamic causality between four pairs (CO2-DF, CO2-TO, CO2-IND, and CO2-TEC), including both 5% and 10% critical values. Beginning with the CO2-DF pair, we observe that there is a two-way causality between financial development and CO2 emissions. Importantly, a significant Granger causality runs from CO2 to DF and vice versa over many periods, including 2005 and 2018. In other words, this finding demonstrates that financial development increases CO2 emissions in Thailand. Given Thailand’s reliance on the oil industry for most of its economic growth, it is not surprising that the country’s banking sector is consuming more energy from fossil fuels to support its operations. In addition, financial growth increases income and eases credit restrictions, hence promoting infrastructure development initiatives. In this economy, all of these elements have the potential to increase energy consumption and CO2 emissions. These outcomes are in line with Lv and Li [5], Khezri et al. [36], Bui [19], and Kihombo et al. [37].

As for the time-varying Granger causality between trade openness and CO2, the result provides evidence that the two-way causality is detected, covering the period from 1998, 2014, and 2016–2022. The longest period of time-varying causality is found in recent years, which may be due to increased TO as a tool for economic development. TO that seeks to boost economic growth is another explanation for the shift in the causal association observed during the second half of the study period. Trade channels have a significant impact on energy prices, particularly oil prices, which leads to a high degree of environmental degradation in this country. Further, ThaiLan started focusing more on exports in the 1990s, and administration and policies for exports have significantly improved. The governments’ liberalization policies throughout the first part of the decade supported the flow of inputs for local and export-oriented businesses, which in turn caused a boom in imports relating to consumers and non-production. This demonstrates that Thailand prioritized exports over the environment when implementing policies. Our results are generally in line with Ertugrul et al. [25], Zhang et al. [26], and Le et al. [46], who reveal a significant causality between CO2 emissions and trade openness.

Looking now at the industrialization causality impact over CO2 emissions, this considerable causal nexus between both variables is more visible under the REC specification during the sample shown. The Wald statistic sequence always exceeds its corresponding 10% significance level except for the periods of 2010–2012. Taking into account the CO2 emissions causality impact over industrialization, a specific period of highly significant causality can be determined between 2000 and 2021. This implies that increasing industrialization has a considerable influence on the CO2 in Thailand. The result uncovers that when IND is rising in this nation, the environment is more likely to be negatively impacted. The findings have theoretical significance because Shahbaz et al. [58], Mentel et al. [12], Mahmood et al. [35], and many more revealed how technological innovations and industrial growth can result in higher emissions rates, which gradually negatively impact the environment. Our findings are also supported by other studies; for example, Musa et al. [28] and Xu and Lin [4] uncover that industrialization negatively impacts environmental quality.

Furthermore, we observe a bidirectional causality in the case of the CO2-TEC pair; there is evidence of significant causal linkages between TEC and CO2 emissions for the full period. Specifically, we detect a unidirectional causality running from CO2 to TEC at different time sequences between 1998 and 2022. This means that increased use of advanced technology by individuals harms the environment by causing significant emissions. Put differently, technological innovation can predict CO2 emissions, which has significant policy implications for policymakers in Thailand. This outcome confirms the studies of Yu and Du [39], Ma et al. [41], Wang and Zhu [42], and Jian and Afshan [14] that investigate the interplay between technological innovations and emissions in different countries. Nevertheless, the result contradicts the articles of Bilal et al. [15] for international countries, and Adebayo et al. [43] for Portugal, who documented that technological innovation enhances environmental quality.

In addition, both TEC and IND exhibit a substantial bidirectional Granger association with CO2 emissions, albeit not throughout the sample period. Compared to TO and DF, the causal effect of TEC and IND on CO2 emissions suggests that environmental quality shifts from energy and carbon-intensive economies to decarbonised economies could play a key role in this country’s attempts to ameliorate climate change. As a result, we advocate a gradual transition from nonrenewable to renewable energy technologies as increased intermittent, energy stability, sustainable growth, and pollution reduction can be achieved.

**Fig 5:**
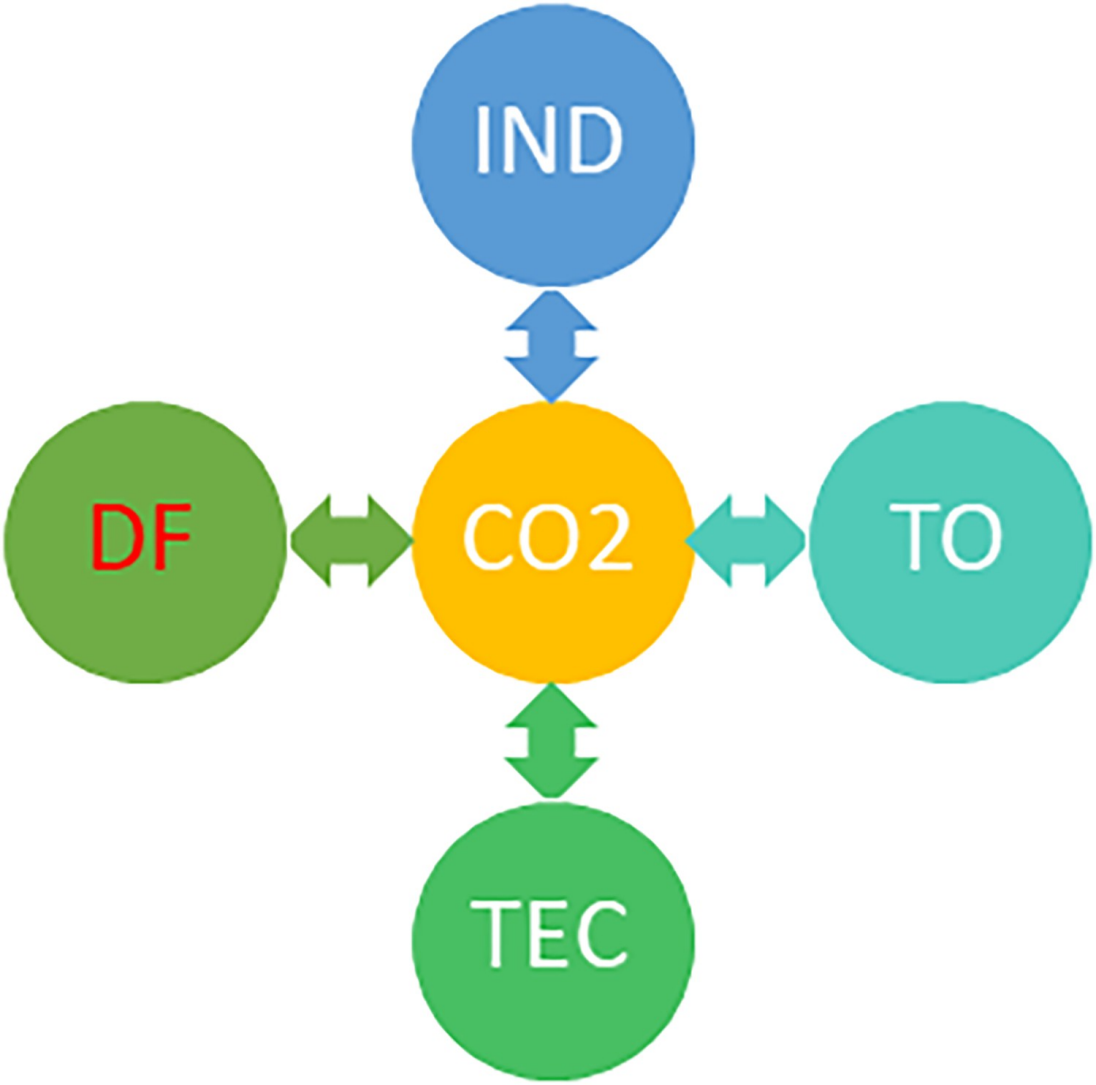
Pairwise causality test results.

The results of the time-varying Granger causality test reveal the persistent bidirectional causality between the selected macroeconomic factors and CO2 emissions. Fig 5 depicts the direction of causality among indicators through a flowchart diagram. This outcome is unsurprising, as Thailand’s economic growth propels the country towards industrialization and encourages excessive energy consumption. Consequently, this process contributes to environmental pollution and depletion of natural resources. These findings align with those of previous studies conducted by Rehman et al. [29], Liu and Bae [34], Khan and Ozturk [18], Cheng et al. [6], and Saidi and Mbarek [50].

In general, our results show that the link between DF, TO, IND, TEC, and CO2 emissions varies over time. Nevertheless, they coincide with empirical data demonstrating the robust influence of IND, DF, TO, and TEC on CO2 emissions between 2012 and 2018, with a notable rise in CO2 emissions per person between 2.75 metric tons in 1999 and 4.06 metric tons in 2018. Consequently, when compared to other periods, there is an exceptionally high level of causality between the variables during these times. Furthermore, DF and IND display a causal relationship with CO2 emissions, although its weak significance (10% level) makes this finding less reliable. This suggests that IND, DF, TO and TEC in Thailand are conductive to boosting the environmental degradation, which implies that higher IND, DF, TO and TEC in Thailand result in higher CO2 emissions.

Identifying time-varying causality between DF, TO, IND, TEC, and CO2 emissions is crucial for policy. If anything, it means that policies aimed at promoting environmentally friendly and sustainable economic growth should not remain static. Put it in another way, our empirical findings suggest that specific policies aimed at promoting growth and mitigating environmental degradation through carbon dioxide abatement must be flexible in response to country structural changes. Otherwise, incorrect conclusions are made. Comparing the linear Granger causality test the Toda-Yamamoto and time-varying causality results for Thailand sheds light on this. The outcomes of the time-varying Granger causality tests uncover three arguments below:

- The Granger causality test assumes a consistent relationship between DF, TO, TEC, IND, and CO2 emissions throughout the sample period, which is not the case.
- There is a time-varying causal relationship between the interested pairs.
- Granger causality tests clearly do not produce healthy results in circumstances when the causal relationship changes over time, when the causal relationship is unstable.

## 6. Conclusion

Many studies investigate the role of economic growth and technological innovation in driving CO2 emissions. However, most of these articles focus on advanced economies, leaving a gap in high-quality research within the Thai context. The aim of such studies is to develop evidence-based policies that can help mitigate the effects of climate change. Therefore, this study provides a thorough analysis of the impact of financial development, industrialization, trade openness, and technological innovation on environmental degradation in Thailand from 1991 to 2022. The research employs novel time-varying Granger causality tests to evaluate and illustrate the directional relationship between the indicators.

Based on our analyses, we initially employ the traditional Toda-Yamamoto causality test. The results indicate a two-way causality between financial development and CO2 emissions, as well as a unidirectional causality from industrialization to CO2 emissions in Thailand. Secondly, we observe that the causality between the examined variables is not consistently evident. In other words, the Granger causal nexus between indicators varies over time and differs across various macroeconomic factors. Overall, there exists a bidirectional relationship between financial development, industrialization, trade openness, technological innovation, and CO2 emissions across different time periods.

The time-varying causality outcomes reveal significant changes in associations over time, contrasting with the static causality indicated by the Toda-Yamamoto test among the four interactions. Put differently, there exists a dynamic causal link between industrialization, financial development, trade openness, technological innovation, and CO2 emissions. These findings have implications for both policy and research. The results of the dynamic causality methodology suggest that policies aimed at fostering environmentally friendly and sustainable economic development in Thailand need to be adaptable, considering the economic and structural changes occurring in the country.

## 7. Policy insights

The empirical results suggest several policy implications that can assist authorities or policymakers in Thailand in addressing the negative environmental effects of economic development. First, Thailand should implement targeted approaches to reduce industrial CO2 emissions at different stages of industrialization, given the statistically significant time-varying Granger causality between industrialization and emissions. Additionally, Thailand could actively explore alternative energy sources and promptly adopt sophisticated technology and equipment from other nations to effectively decrease total CO2 emissions.

Second, there is a robust correlation between financial development and CO2 emissions, indicating that the detrimental effects of financial development on environmental quality surpass the beneficial ones. These findings advocate for several measures to mitigate the environmental impact of financial development. To enhance the positive outcomes of financial development, government interventions should focus on implementing energy-efficient policies. Policy makers might contemplate advancing the financial sector alongside crafting environmental protection policies. This could involve overseeing financial institutions and offering loan opportunities for environmentally friendly and carbon-neutral projects. Additionally, it may be prudent to take into account the repercussions of adverse shocks in financial development when shaping financial sector policies.

Third, there was confirmation of a two-way short-run causal linkage between trade openness and emissions, suggesting a mutual dependence between the two metrics. Therefore, in order to promote sustainable economic development in this nation, the government should adopt green trade policies and step-up initiatives related to trade. Governments should support environmental laws related to trade and encourage the adoption of clean, green technologies in the manufacturing of traded goods. As international commerce liberalizes, increasing the participation of the tertiary industry in foreign direct investment is a feasible approach to help reduce pollution. Furthermore, the present paper recommends that regulators seek regional cooperation in the process of formulating policies related to multilateral trade. Policymakers should consider reducing trade openness during economic cycles to mitigate emissions from "dirty products" traded.

Last, to attain both heightened productivity and greener domestic consumption, the promotion of technical innovation is imperative. Governments should persist in bolstering their collaboration with developed nations leading in technical advancements and augment funding for Research and Development (R&D) endeavors to generate cutting-edge inventions. This will aid Thailand in mastering clean-environment technologies, as elevated emissions in developing nations could exert a detrimental global impact on environmental quality.

The present study has its limitations. Firstly, the use of CO2 emissions as a proxy for environmental degradation may not fully capture the breadth of the issue, which also encompasses factors such as Ecological Footprint or load capability factor. Additionally, important factors such as urbanization, green technology, and government spending on emissions have not been considered in this research. These gaps present opportunities for future scholarship to address, particularly in the context of Thailand.
