# Correction: Time-varying causality relationships between trade openness, technological innovation, industrialization, financial development, and carbon emissions in Thailand

**DOI:** 10.1371/journal.pone.0308342

**Published:** 2024-07-31

**Authors:** Nguyen Thi Quy, Nguyen Chi Hai, Ha Thi Thieu Dao

The funding statement for this paper is incorrect. The correct funding statement is: This research is funded by the University of Economics and Law, Ho Chi Minh City, Vietnam, and Vietnam National University, Ho Chi Minh City, Vietnam.
